# What makes you can also break you: mitochondrial permeability transition pore formation by the c subunit of the F_1_F_0_ ATP-synthase?

**DOI:** 10.3389/fonc.2013.00025

**Published:** 2013-02-19

**Authors:** Christos Chinopoulos, Gyorgy Szabadkai

**Affiliations:** ^1^Department of Medical Biochemistry, Semmelweis UniversityBudapest, Hungary; ^2^Department of Cell and Developmental Biology, Consortium for Mitochondrial Research, University College LondonLondon, UK; ^3^Department of Biomedical Sciences, University of PaduaPadua, Italy

A number of cellular stresses and cytotoxic agents trigger mitochondrial permeability transition (mPT), considered as a final common pathway of cell death (Brenner and Grimm, 2006). mPT follows the formation of a large non-selective pore (mPTP) in the inner membrane of mitochondria (IMM), permeable to molecules up to 1.5 kD. Accordingly, mPT leads to metabolic insufficiency of mitochondria (Chinopoulos and Adam-Vizi, 2010) and has been assumed to underlie the induction of both accidental (necrotic) and regulated forms of cell death (e.g. the intrinsic apoptotic or necroptotic pathways) (Galluzzi et al., 2012). Indeed, pharmacological and posttranslational modification of cyclophilin-D (cypD), an established regulatory subunit of the mPTP, has been shown to modulate cell sensitivity to death induction in several pathologies (Giorgio et al., 2011). Thus, the pore will likely represent the target of a novel regulated cell death modality but the lack of information on its molecular identity is persistently impeding the characterization of this pathologically highly relevant pathway.

Recently, cypD has been shown to interact with and regulate the F_1_F_0_ ATP-synthase, the main molecular motor of chemiosmotic ATP production in the mitochondrion (Giorgio et al., 2009; Chinopoulos et al., 2011), raising the odd suspicion that the most fundamental pillar of cellular energy metabolism might also be the gatekeeper, or even the principal component of mPT (Chinopoulos and Adam-Vizi, 2012). The first experimental trial of this curious idea has now emerged in the latest issue of Cell Cycle (Bonora et al., 2013). The study addressed the effect of the overexpression and silencing of the ring-forming subunit c of the membrane spanning F_0_ unit, and demonstrated that the propensity of mPT highly correlates with the subunit c expression levels. These results imply that a conformational change of the c-ring might transform it to a non-selective pore, presenting a provocative idea leading to a series of outstanding questions.

The first, conceptually most challenging problem is how to separate the ATP-synthase and eventual pore-forming activity of the F_1_F_0_ complex in the experimental design. The rotation of the membrane-embedded ring formed by the c subunits of the ATP-synthase is driven by the proton motive force across the IMM. Since, according to the current model, the protonation/de-protonation cycle of each c subunit is required for the translocation of one H^+^, and a complete 360° rotation of the ring generates 3 ATP molecules on the α catalytic subunit, the number of c subunits will dictate the bioenergetic cost of making one ATP per F_1_F_0_ ATP-synthase (Watt et al., 2010). Knockdown of the c subunit can either decrease the number of c subunits per F_1_F_0_ ATP-synthase molecule, or reduce the overall number of functional F_1_F_0_ ATP-synthases, but in both cases it would alter the efficiency of the ATP hydrolysis or production at a given mitochondrial membrane potential. Thus, separating the consequent bioenergetic effects from the direct molecular consequences of c subunit knockdown is essentially unworkable. The authors addressed this issue by silencing the catalytic F1-localized α subunit as a control, which had no effect on mPT, while the genetic manipulation should have had the same impact on the number of functional F_1_F_0_ ATP-synthases. This indicates that the observed effect on mPT is indeed specific to the c subunit, but the detailed characterization of an eventually altered stoichiometry of the F_1_F_0_ complex on the ATP/H^+^ (or the P/O) ratio warrants further analysis, particularly in view of the fact that the number of copies of subunit c is constant and appears to be independent of the metabolic state within all vertebrate animals and most invertebrate species (Watt et al., 2010).

The second, technically challenging question is whether the F_0_ c-ring can form a pore with characteristics of the mPTP? Would other subunits also be required? The idea might not be too far-fetched, since it has been shown that subunit c, reconstituted in lipid bilayers, forms a voltage sensitive pore mediating a Ca^2+^-regulated cation current (McGeoch et al., 2000). Moreover, elastic network modeling inferred that the c-ring exhibits significant flexibility allowing for some extreme deformations during operation of the F_1_F_0_ ATP-synthase (Saroussi et al., 2012). Again, whether an mPTP-like pore can be formed this way, and if yes, under what conditions/composition will need further rigorous testing.

Finally, these findings recall an old question about the pharmacology of mPT. Oligomycin, a potent inhibitor of the F_1_F_0_ ATP-synthase, targets the c subunit, suggesting that it might also affect mPTP formation. Whilst many early studies on isolated mitochondria have shown mPT inhibition by oligomycin, the effect was always accounted for changes in ATP/ADP concentrations, locking the pore in a closed conformation. We are aware only one study so far addressing the direct effect of oligomycin on mPT-induced cell death. Shchepina et al. have shown that whilst oligomycin (acting on the F_0_ c subunits) was able to almost completely block TNF/emetine-induced cell death, aurovertin B (inhibiting ATP synthesis on the F_1_ catalytic subunits) had no effect on mPT in this cell death modality (Shchepina et al., 2002). This result echoes the findings of Bonora et al. (2013) but yet again provokes further questions. First, can the mPTP forming abilities of the c subunits be specifically targeted without compromising cell viability? Second, while oligomycin efficiently blocked TNF/emetin-induced cell death, it was ineffective against staurosporine-induced apoptosis, suggesting the existence of a specific oligomycin-sensitive (c subunit-mediated) cell death modality. Will this modality fit into the catalogue of the known biochemically characterized cell death pathways (e.g., necroptosis) or will it represent an entirely novel death subroutine?

Altogether, as shown by the above list of outstanding questions, we anticipate that the findings by Bonora et al. (2013) are only the beginning of a new shake-up in the mitochondrial field, and more detailed analyses of the role of the F_1_F_0_ ATP-synthase in mPT and cell death will soon follow.
